# Roundtable discussion: how the World Hepatitis Summit 2015 strengthened stakeholders’ efforts to combat viral hepatitis

**DOI:** 10.1186/s41124-016-0018-4

**Published:** 2016-10-07

**Authors:** Charles Gore, Stefan Wiktor, David Goldberg, Sharon Hutchinson, Jose Antonio Oñate Moreno, Raquel Peck

**Affiliations:** 1 World Hepatitis Alliance 1 Baden Place, London, SE1 1YW UK; 2 World Health Organization, Geneva, Switzerland; 3 Health Protection Scotland, Glasgow, UK; 4 Glasgow Caledonian University, Glasgow, UK; 5 Fundación Hepatos Aión, AC, Mexico City, Mexico

**Keywords:** Hepatitis, Civil Society, Viral Hepatitis, Health Protection, Patient Organization

## Abstract

The inaugural World Hepatitis Summit was jointly convened by the World Health Organization (WHO) and the World Hepatitis Alliance and hosted by the Scottish Government and supported by Glasgow Caledonian University and Health Protection Scotland in September 2015. The three day event convened a broad range of stakeholders to meet and share ideas, experience and best practice in addressing the many facets of viral hepatitis prevention, diagnosis and treatment.

With the next World Hepatitis Summit scheduled to take place from 1 to 3 November 2017, the World Hepatitis Alliance asked *Hepatology, Medicine and Policy* to commission a roundtable discussion article in order to encourage reflection on how the 2015 Summit was significant for stakeholders’ efforts and why it is important to keep the momentum going ahead of the World Hepatitis Summit 2017 and in the light of the newly adopted first-ever Global Health Sector Strategy on Viral Hepatitis.

## Background

### Charles Gore, President of the World Hepatitis Alliance

The inaugural World Hepatitis Summit was jointly convened by the World Health Organization (WHO) and the World Hepatitis Alliance (WHA) and hosted by the Scottish Government and supported by Glasgow Caledonian University and Health Protection Scotland in September 2015. It was the first opportunity for a broad range of policy-makers and patients to come together to discuss the global response to viral hepatitis.

During three days of plenary sessions, panel discussions, simulation exercises and other events, participants focused on the central question of how to strengthen efforts to address viral hepatitis at the national level through comprehensive national plans in line with World Health Assembly Resolution 67.6. At the closing session, delegates endorsed the Glasgow Declaration on Viral Hepatitis, which calls on governments to develop and implement comprehensive national hepatitis plans to drive action toward the elimination of viral hepatitis as a public health concern (Table 1).

**Table 1:** The Glasgow Declaration on Viral Hepatitis

*Because there are 400 million people living with hepatitis B or hepatitis C infection with no country/region unaffected,* *Because there is a lack of global awareness and most persons with hepatitis remain undiagnosed,* *Because 1.4 million people die every year from complications of viral hepatitis yet most of these deaths can be prevented,* *Because there are highly effective measures to prevent new hepatitis B and C infections and highly effective treatments that can suppress hepatitis B virus replication and cure hepatitis C infection,* *Because universal access to prevention, diagnosis, care and treatment is a human right and promoting access to and affordability of these services is the responsibility of all stakeholders,* The participants of the inaugural World Hepatitis Summit believe it is possible and essential to set as a goal the elimination of both hepatitis B and C as public health concerns. We therefore call upon governments in all jurisdictions to develop and implement comprehensive, funded national hepatitis plans and programmes in partnership with all stakeholders and in line with the World Health Assembly Resolution 67.6 and, in collaboration with the World Health Organization, to define and agree on realistic yet aspirational global targets for prevention, testing, diagnosis, care and treatment.	

With the next World Hepatitis Summit scheduled to take place from 1 to 3 November 2017, it will be critical once again to bring together a wide range of stakeholders to accelerate action toward elimination. The World Hepatitis Alliance asked *Hepatology, Medicine and Policy* to commission this roundtable discussion article in order to encourage reflection on how the 2015 Summit was significant for stakeholders’ efforts and why it is important to keep the momentum going ahead of the World Hepatitis Summit 2017. We are grateful to the diverse group of World Hepatitis Summit 2015 delegates who responded to the Alliance’s invitation to write roundtable contributions.

## World delegates initiate a global response to hepatitis

### Stefan Wiktor, Team Lead, Global Hepatitis Programme, World Health Organization

The World Hepatitis Summit in 2015 was the inaugural gathering of over 500 stakeholders. Key representatives, including policy-makers, patient groups, civil society, funders, public health specialists and others, contributed to the global hepatitis discourse during the broad array of sessions offered. By bringing together these diverse groups, the Summit was able to promote collaboration in the efforts to combat viral hepatitis—an approach that is aligned with WHO’s aim to achieve equitable and accessible hepatitis services for all. In particular, patient involvement helped to define the scope of the Summit and was seen as crucial to its success.

Importantly, the Summit proved to be an ideal venue for the release of the Glasgow Declaration on Viral Hepatitis which initiated a momentum amongst patients and WHO Member States, which was strengthened by the extensive stakeholder consultations around WHO’s Global Health Sector Strategy on Viral Hepatitis (GHSS). The GHSS, which was adopted at the World Health Assembly in May 2016, supports this overall vision and outlines a set of targets and strategic priorities to meet the goal.

The Glasgow meeting also provided an opportunity for WHO to showcase and enable the implementation of the normative guidance and technical assistance offered by different teams and departments within WHO Headquarters and the Regional Offices. As the theme of the Summit was “National Planning for Viral Hepatitis,” the Summit directly supported the World Health Assembly resolution 67.6, which calls on WHO “to provide the necessary technical support to enable Member States to develop robust national viral hepatitis prevention, diagnosis and treatment strategies.”

The Summit was successful in raising the visibility of viral hepatitis and increasing awareness among Member State policy-makers of the importance of a comprehensive response. It also served as an effective learning forum, providing many of the tools necessary for national planning for viral hepatitis.

## Patients and policy-makers unite to eliminate viral hepatitis

***“But the importance of good data in making the odyssey a success should not be underestimated”***

### Professors David Goldberg and Sharon Hutchinson, Health Protection Scotland and Glasgow Caledonian University

The first World Hepatitis Summit was a historic event whose impact will become apparent in the future. A brand has been established, a declaration to eliminate hepatitis as a serious public health concern has been voiced. A statement has been made about the importance of civil society and politicians collaborating in the process of service development and delivery. But what next?

The Summit represents a starting point—a platform, a foundation, on which WHO and WHA can build a highly visible movement to call attention to and promote the miraculous advances in hepatitis B and C therapeutics and primary prevention interventions. But the Summit also reminded us that more needs to be done and that a coordinated effort is vital. Future Summits, to be held every two years, should serve as a key opportunity to bring a wide range of stakeholders together and track progress made in improving hepatitis services.

Because the Summit is about civil society, patients, public health specialists and policy-makers, and not just the latter, it is well placed to do this job. Patient (representative) power, in particular, as demonstrated in Portugal and Scotland in relation to hepatitis C in 2007 and 2015, respectively, might ultimately be the decisive factor in making governments act. But without reliable process and outcome data, both patients and policy-makers will find it difficult to make the case for such investment.

Obtaining serial outcome data on the number of people presenting with, or dying from, end stage liver disease is crucial to identify the seriousness, scale and trajectory of the problem, and to assess the intensity and type of intervention(s) required to address it. As observed in Scotland during 2007–08, politicians and civil servants became much more engaged when confronted with observed data on life-threatening disease and death than with estimated data on the number of people with HCV infection.

Securing serial process data on the number and type (especially from a liver disease stage perspective) of people treated with anti-viral therapy is vital if a strategic approach to morbidity and mortality reduction is to be achieved. For example, in the context of limited healthcare resources and expensive therapies, intelligent, prioritized prescribing—focusing on those at greatest risk to health—will likely lead to a faster reduction in such serious outcomes than the converse. The World Health Organization’s Global Health Sector Strategy underpinned by 2021 and 2030 elimination targets (adopted in May 2016), will undoubtedly advance the cause; however, currently, few countries are in an adequately data-rich position to calibrate a proportionate response consistent with WHO’s elimination vision. This is because of insufficient resources and/or a failure to fully appreciate the important role of surveillance/monitoring tools in optimizing the efficiency of deploying government funds to improve the health of the population as a whole. Nevertheless, 2016 should be for hepatitis B and C and associated end-stage disease what 1997 was for HIV and AIDS—a signal year indicating that, at last, we are on the road to disease elimination. The World Hepatitis Summit is a start toward that goal.

## World Hepatitis Summit strengthens patient power

### Jose Antonio Oñate Moreno, Director, Fundación Hepatos Aión, AC

Humanity is facing a great challenge, which can be solved effectively only through global collaboration: the control and elimination of viral hepatitis, particularly hepatitis B and C. Nowhere has the urgency of this challenge been highlighted so clearly as at the first World Hepatitis Summit. For patients and policy-makers alike, this event underscored the need for immediate, and united, action to confront and slow the spread of viral hepatitis.

The involvement of patient organizations demonstrated the important consideration that the medical community needs to give to hepatitis patients—those whose lives have been greatly transformed by their experiences living with the virus. The Summit provided these organizations an opportunity to speak on behalf of those who too frequently have been left out of crucial hepatitis conversations and who are now vital partners in helping create plans for effective prevention, treatment and care.

A number of Latin American patient organizations were in attendance and benefited from networking and exchanging ideas with other WHA member organizations. Topics were wide-ranging, from local and regional strategies and best practices, to ways of partnering on initiatives. They also consolidated their work through their regional network, Hepared Latinoamerica. Those of us from Mexico, where hepatitis takes the lives of over 11,000 people annually, agreed to strengthen collaboration with local and regional health authorities to implement a national strategy in 2016 to address viral hepatitis. We also resolved to ensure that patients have access to affordable direct-acting antivirals for hepatitis C. The benefits of attending the conference have become even more apparent to us in the past year.

United by a common goal, all participants at the Summit benefited from the unique and very potent opportunity to interact with others in attendance. The participation of patient organizations, representing the interests of those most directly affected by viral hepatitis, was instrumental in making their experiences and needs known. Their contributions to the global discussion are vital to helping eventually eliminate hepatitis B and C. Ongoing partnerships among health authorities, medical societies and civil service and patient organizations at a local level will help ensure global success in efforts to benefit all patients worldwide.

## The beginning of the end of viral hepatitis

### Raquel Peck, Chief Executive Officer, World Hepatitis Alliance

The World Hepatitis Summit marked a pivotal moment on the road to eliminating viral hepatitis. For the first time, patients and policy-makers joined together to accelerate the global and national response to viral hepatitis. The result: the Glasgow Declaration on Viral Hepatitis, which calls for comprehensive national plans to drive action toward the elimination of viral hepatitis.

The success of the Summit centered on the involvement of patients. Ahead of the official Summit, WHA organized a pre-summit meeting for their members. This was a momentous occasion, as for the first time, our members— many of which are small and under-resourced—were able to meet each other, share experiences and feel part of a wider, global network.

Patient groups compel us to confront the very human side of hepatitis—the physical, emotional and financial tolls that alter the lives of patients, those closest to them and others in their communities. Patients’ personal insights are crucial in helping policy-makers, service providers and others understand the full impact of hepatitis and in helping clarify the range of issues involved in disease prevention and management. Other delegates in attendance, such as those from the World Health Organization and national ministries of health, had the unique opportunity in a public forum to see disease control concepts and health policy objectives rigorously assessed from the perspective of lived experience.

The Glasgow Declaration was one of the most important outcomes of the Summit because it cemented delegates’ commitment to the goal of eliminating hepatitis B and hepatitis C as a public health concern. The striking enthusiasm with which delegates welcomed the Declaration signified a collective determination and a growing appetite in the hepatitis community for more coordinated and collaborative efforts toward the goal of elimination.

This enthusiasm and commitment has continued among all stakeholders since the Summit. Member States adopted WHO’s first ever Global Health Sector Strategy on viral hepatitis (GHSS) in May 2016, with its goal of elimination. Then, on July 28, World Hepatitis Day 2016, the hepatitis community launched NOhep, a global movement to eliminate viral hepatitis by 2030. Additionally, the World Hepatitis Summit 2017 is being developed under the theme of implementing the GHSS. Through continued commitment, shared determination and collective action, we can reach the goal of elimination by 2030.

## Conclusions

The inaugural World Hepatitis Summit, a three day public event jointly convened by the World Health Organization (WHO) and the World Hepatitis Alliance (WHA) and hosted by the Scottish Government and supported by Glasgow Caledonian University and Health Protection Scotland, was a significant event for the hepatitis community. A one of its kind meeting which took place in Glasgow in September 2015, the Summit brought together civil society, patients, public health specialists, policy-makers, and other key stakeholders and provided a collaborative forum to advance the viral hepatitis agenda. Given the success of this initiative it was decided that the Summit will be run every two years by WHA and WHO in collaboration with a different government each time (the next World Hepatitis Summit is scheduled to take place from 1-3 November 2017 in Sao Paulo). In the light of the newly adopted first-ever Global Health Sector Strategy on viral hepatitis, which sets a goal of eliminating these diseases by 2030, and having in mind that to deliver elimination a coordinated effort is needed, we concluded the World Hepatitis Summit is key to accelerate action towards that goal.

